# In Search for the Avian Trigeminal Magnetic Sensor: Distribution of Peripheral and Central Terminals of Ophthalmic Sensory Neurons in the Night-Migratory Eurasian Blackcap (*Sylvia atricapilla*)

**DOI:** 10.3389/fnana.2022.853401

**Published:** 2022-03-07

**Authors:** Katrin Haase, Isabelle Musielak, Leonie Warmuth-Moles, Bo Leberecht, Anna Zolotareva, Henrik Mouritsen, Dominik Heyers

**Affiliations:** ^1^AG Neurosensorik, Institute of Biology and Environmental Sciences, Carl von Ossietzky Universität Oldenburg, Oldenburg, Germany; ^2^Biological Station Rybachy, Zoological Institute of Russian Academy of Sciences, St Petersburg, Russia; ^3^Research Centre for Neurosensory Sciences, Carl von Ossietzky Universität Oldenburg, Oldenburg, Germany

**Keywords:** magnetoreception, neuroanatomy, somatosensory system, songbird, trigeminal system

## Abstract

In night-migratory songbirds, neurobiological and behavioral evidence suggest the existence of a magnetic sense associated with the ophthalmic branch of the trigeminal nerve (V1), possibly providing magnetic positional information. Curiously, neither the unequivocal existence, structural nature, nor the exact location of any sensory structure has been revealed to date. Here, we used neuronal tract tracing to map both the innervation fields in the upper beak and the detailed trigeminal brainstem terminations of the medial and lateral V1 subbranches in the night-migratory Eurasian Blackcap (*Sylvia atricapilla*). The medial V1 subbranch takes its course along the ventral part of the upper beak to innervate subepidermal layers and the mucosa of the nasal cavity, whereas the lateral V1 subbranch runs along dorsolateral levels until the nostrils to innervate mainly the skin of the upper beak. In the trigeminal brainstem, medial V1 terminals innervate both the dorsal part and the ventral, magnetically activated part of the principal sensory trigeminal brainstem nuclei (PrV). In contrast, the lateral V1 subbranch innervates only a small part of the ventral PrV. The spinal sensory trigeminal brainstem nuclei (SpV) receive topographically ordered projections. The medial V1 subbranch mainly innervates rostral and medial parts of SpV, whereas the lateral V1 subbranch mainly innervates the lateral and caudal parts of SpV. The present findings could provide valuable information for further analysis of the trigeminal magnetic sense of birds.

## Introduction

In birds, the perception of somatosensory information from the facial/beak region is of central importance for a wide range of behaviors. To mediate them, their underlying trigeminal sensory system has undergone a particularly high degree of diversification. In addition to mechanoreception, proprio-, thermo-, chemo-, and nociception, the birds’ trigeminally mediated behavioral repertoire ranges from feeding, tactile exploration of the environment, hatching, grooming, climbing, and nest building (Wild, 2015; Faunes and Wild, 2017b) to cognitively complex skills such as skillful food handling (Ziswiler, 1965; Zweers et al., 1994) and/or the use of tools (Weir et al., 2002).

Trigeminal sensory information is transmitted *via* the fifth cranial, i.e., trigeminal nerve (N. V.), toward the brain. N. V. splits into ophthalmic (V1), maxillary (V2), and mandibular (V3) branches (Bubien-Waluszewska, 1981), all of which fuse in the trigeminal (Gasserian) ganglion to enter the brain at the level of the rostral pons. Toward the brain, N. V. splits into an ascending and a descending trigeminal tract to terminate in the principal (PrV) and spinal (SpV) sensory trigeminal brainstem nuclei, respectively (Wild and Zeigler, 1996). SpV, from rostral to caudal, can be subdivided into an oral, interpolar, and caudal part. SpV has projections within SpV, and projects to PrV (Arends et al., 1984; Faunes and Wild, 2017a). PrV consists of an oval-shaped dorsal and a ventrally attached, crescent-shaped part. These subparts relay trigeminal information to different parts of the forebrain (Wild et al., 1985; Wild and Farabaugh, 1996; Wild and Zeigler, 1996; Mouritsen et al., 2016; Kobylkov et al., 2020).

Some birds are also among nature’s foremost navigators (Alerstam, 1993; Wiltschko and Wiltschko, 1995; Frost and Mouritsen, 2006; Mouritsen et al., 2016; Chernetsov, 2017; Chernetsov et al., 2017; Heyers et al., 2017; Mouritsen, 2018; Wynn et al., 2022). Accumulating evidence suggests that the trigeminal system, in particular V1, is also involved in the perception of geomagnetic information in night-migratory songbirds. Night-migratory songbirds, which can compensate for both geographical and virtual magnetic displacements by correcting their migratory direction (Chernetsov et al., 2008, 2017; Kishkinev et al., 2015, 2021), failed to do so when their V1s were surgically ablated. Instead, they headed in the same migratory direction as before the displacement (Kishkinev et al., 2013; Pakhomov et al., 2018). This indicates that V1 is involved in sensing magnetic positional information. On the brain level, the ventral part of PrV and the SpV were shown to display significantly increased neuronal activation when the birds were exposed to a strongly changing magnetic field stimulus. Both compensation of the ambient magnetic field or V1 ablation significantly decreased the number of magnetically activated neurons (Heyers et al., 2010; Lefeldt et al., 2014; Elbers et al., 2017). Unlike the dorsal part of PrV, which sends somatosensory information *via* the quintofrontal tract to the telencephalic nucleus basorostralis (Wild et al., 1985; Wild and Farabaugh, 1996; Wild and Zeigler, 1996; Kobylkov et al., 2020), the magnetically activated ventral part of PrV was recently shown to form the origin of a morphologically distinct neuronal population exclusively connected to the telencephalic frontal nidopallium. The ventral PrV part could thus represent part of a brain pathway specifically dedicated to transmitting magnetic map information to multisensory integration centers in the avian forebrain (Kobylkov et al., 2020).

In stark contrast to the growing body of evidence for V1-mediated magnetoreception and its central nervous representation, many attempts failed to find an underlying sensory structure (Williams and Wild, 2001; Fleissner et al., 2003, 2007; Falkenberg et al., 2010; Mouritsen, 2012; Treiber et al., 2012, 2013; Engels et al., 2018). Most of these studies used generic neuronal markers to label nerve-fiber terminals potentially harboring the magnetic sensor. One aspect which has only been sparsely investigated in this context is the detailed course of V1 up to its distal terminals in the upper beak. In fact, this has only been described in non-migratory chicken (Bubien-Waluszewska, 1981). Furthermore, while the V1 terminations in the trigeminal brainstem complex have been described in different species (Wild and Zeigler, 1996; Heyers et al., 2010; Lefeldt et al., 2014; Elbers et al., 2017; Faunes and Wild, 2017a,b; Kobylkov et al., 2020), these studies did not differentiate between terminations originating from different V1 subbranches, namely, the medial and lateral V1 subbranch. Assuming that any magnetic sensory structure should be located within or in the near vicinity of V1 fiber terminals, the specific mapping of the medial and lateral V1 subbranches in a night-migratory songbird could narrow the regions to be searched for the elusive trigeminal magnetic sensors.

Therefore, the aim of the present study was to perform selective neuronal tract-tracing of the medial and lateral V1 subbranches in the long-distance night-migratory songbird Eurasian blackcap (*Sylvia atricapilla*) to map their respective courses from their distal nerve fiber terminals within the upper beak up to their projections in the trigeminal brainstem complex.

## Materials and Methods

### Animals

A total of 8 male and 6 female adult Eurasian blackcaps (*S. atricapilla)* were used for this study. All animal procedures were approved by the Animal Care and Use Committees of the Niedersächsisches Landesamt für Verbraucherschutz und Lebensmittelsicherheit (LAVES, Oldenburg, Germany, Az.: 33.19-42502-04-15/1865; 33.19-42502-04-20/3492; 33.8-42502-04-17/2724). The birds were wild-caught using mist nets after the breeding season and during autumn migration in the vicinity of University Oldenburg. The birds were housed in pairs in indoor wire cages (102 cm × 50 cm × 40 cm) at the institute’s animal housing facility. They were kept at around 21°C and were exposed to a light regime simulating the natural circannual and circadian light-dark cycle of Oldenburg. Food and water were provided *ad libitum*.

### Neuronal Tract Tracing

To visualize the course of V1 and its subbranches, neuronal tract tracer was either injected into the medial V1 subbranch, the lateral V1 subbranch, or the entire V1. Two different types of anesthesia were used to allow accessibility to the respective nerve subbranches: for entire V1 tracings, inhalation of anesthesia with 1–1.5% volume isoflurane CP ^®^ (1 ml/ml; cp-pharma, Burgdorf, Germany) dissolved in oxygen and administered through a beak mask was used. For medial and lateral V1 tracings, we administered a mix of ketamine hydrochloride (Ketamin, WDT, Garbsen, Germany) and medetomidine (Domitor ^®^, Orion Pharma, Ismaning, Germany), each at a concentration of 0.1 ml/kg body weight dissolved in 0.9% sodium chloride (NaCl), into the pectoral muscle. For the tracings, the animals were head-fixed. For entire V1 tracings, the nerve was accessed unilaterally through an incision along the dorsal rim of the orbit and careful retraction of the eyeball and oculomotor muscles (five animals). This procedure was identical to previous studies, in which V1 was either tracer-labeled or surgically sectioned (Zapka et al., 2009; Heyers et al., 2010; Kishkinev et al., 2013; Lefeldt et al., 2014; Elbers et al., 2017; Pakhomov et al., 2018). For medial V1 tracings, the subbranch was accessed through a small window cut at the inside of the upper beak at the caudal end of the cleft palate and nostrils (five animals). The lateral V1 subbranch was accessed through a window-cut at the lateral beak base (four animals). A total of 100–300 nl of the neuronal tracer substance Cholera toxin B subunit (CtB; 1% in distilled water; C9903, Sigma-Aldrich, St. Louis, MO, United States) was administered by pressure injections using a microinjector (WPI-2000, World Precision Instruments, Sarasota, FL, United States) and beveled glass capillaries. After the surgery, the skin and tissue were repositioned and resealed using cyanoacrylate surgical glue (Histoacryl ^®^, BRAUN, Rubi, Spain). Meloxicam (Metacam ^®^, Boehringer Ingelheim, Ingelheim, Germany; 0.1 ml/kg body weight dissolved in 0.9% NaCl) was administered to each bird intramuscularly in the pectoral muscle after 24 and 48 h for post-surgical analgesia. The birds were given 5 days to recover from the surgery and to let the tracer be transported.

### Magnetic Stimulation

To show which parts of the trigeminal brainstem complex are magnetically activated, birds were exposed to a magnetic field stimulus. Single birds were placed in a round Plexiglas cage on a wooden table at the center of a double-wrapped, three-axis Merrit four-coil system (Kirschvink, 1992). The coil system was housed in an aluminum-shielded chamber acting as a Faraday cage, which allowed static magnetic fields to pass through, while radiofrequency fields were attenuated (Engels et al., 2014; Schwarze et al., 2016). The three axes of the coil system were powered by three separate constant-current power supplies (BOP 50–4 M, Kepco Inc., Flushing, NY, United States), which were controlled by a custom-written MatLab script (Version: 2013a, Matlab, Mathworks, Natick, MS, United States; Lefeldt et al., 2014). The birds experienced a magnetic stimulus containing randomized small and large variations in two alternating 5-min blocks: in the first 5 min, only the horizontal direction of the magnetic field changed every 30 s by approximately 90°, while inclination (mean ± standard deviation: 67.6 ± 0.8°) and field intensity (48,800 ± 400 nT) only marginally changed. In the alternating 5 min, the field intensity (18,500–111,000 nT), horizontal direction (0–360°), and inclination (−84.9 ± 76.6°) changed randomly and independently every 30 s. This stimulus was identical to the one which was used previously and which consistently resulted in the activation of the trigeminal brainstem complex (Heyers et al., 2010; Lefeldt et al., 2014; Elbers et al., 2017; Kobylkov et al., 2020). The setup was illuminated with light bulbs at a light intensity of approximately 2.5 mW/m^2^ (Zapka et al., 2009). As in previous studies, we tried to minimize any potential brain activation effects caused by excessive mechanical stimulation of the beak or motor activity (Feenders et al., 2008) by monitoring the behavior of each bird in real time using infrared cameras. A bird was only taken for brain analysis when it was constantly awake and sitting still during the magnetic exposure for at least 60–90 min.

### Tissue Processing

Immediately after magnetic exposure, birds were deeply anesthetized with pentobarbital (Narcoren ^®^, Boehringer Ingelheim, Ingelheim, Germany; 2.5 ml/kg body weight) and transcardially perfused with 0.9% NaCl followed by 4% paraformaldehyde (PFA) dissolved in phosphate-buffered saline (PBS; pH 7.4). Brains and beaks were extracted, post-fixed in 4% PFA in PBS for 24 h, and cryoprotected in 30% D(+)-saccharose dissolved in PBS for at least 48 h. Brains and beaks were cut using a freezing microtome (Leica CM 1860, Wetzlar, Germany). Brains were cut in 40-μm thick slices in the frontal plane in six parallel series and the free-floating slices were collected in 0.1% sodium azide dissolved in PBS. Beaks were cut in 25-μm thick slices in the frontal plane in 10 parallel series, dried on gelatinized glass slides (Menzel SuperFrost ^®^ Plus, Thermo Fisher Scientific, Waltham, MA, United States), and stored at −20°C.

### Immunohistochemistry

**FIGURE 1:**
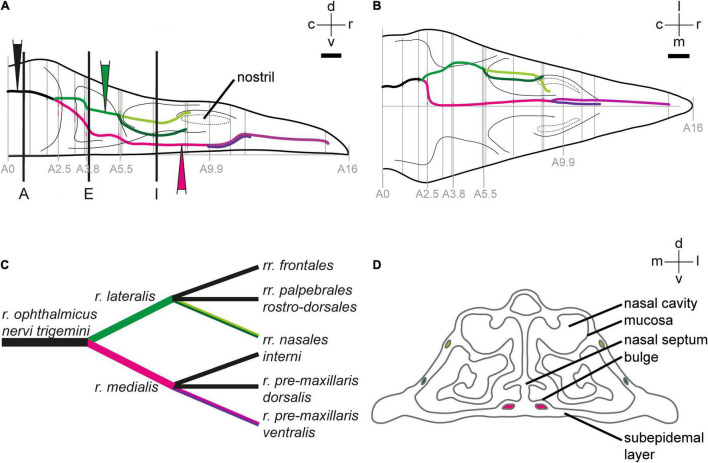
Anatomy of the ophthalmic branch of the trigeminal nerve (V1). 2D-reconstruction of the course of the V1 subbranches in the upper beak of Eurasian blackcaps in side view **(A)** and top view **(B)**. Gray lines indicate the section levels used for the reconstruction. The section level, in which V1 exits the orbit, was set as the zero coordinate (*A* = 0 mm), and the tip of the beak was set as the most rostral coordinate (*A* = 16 mm). Black lines in panel **(A)** indicate the section planes shown in Figure 2. Color-filled triangles in panel **(A)** indicate the injection sites. The lateral V1 subbranch is shown in light/dark green, the medial V1 subbranch in magenta/pink/violet. **(C)** Nomenclature of V1 subbranches according to Bubien-Waluszewska (1981). The subbranches depicted in black in panel **(C)** were described in chicken (Bubien-Waluszewska, 1981), but we could not locate them in the Eurasian blackcap. **(D)** Schematic crosssection through the upper beak at the level of A 7 mm depicts the anatomical terminology of the upper beak. Abbreviations: c, caudal; d, dorsal; l, lateral; m, medial; r, rostral; v, ventral. Scale bars: **(B)** (for **A,B**), 1 mm.

**FIGURE 2:**
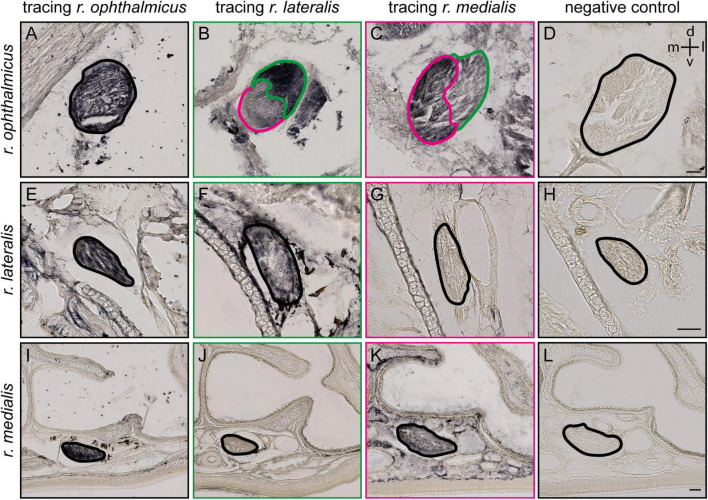
Tracing of the ophthalmic branch of the trigeminal nerve (V1) and its lateral and medial subbranches. **(A,E,I)** Representative frontal slices at different section planes (see black lines in Figure 1A) of the upper beak of Eurasian blackcaps after entire V1 tracing (*r. ophthalmicus*; black), **(B,F,J)** lateral V1 subbranch tracing (*r. lateralis*; green) and **(C,G,K)** medial V1 subbranch tracing (*r. medialis*; magenta) with Cholera toxin B subunit. Selective tracing of either the medial or lateral V1 subbranch that was confirmed as only the medial subbranch but not the lateral one is labeled in medial V1 tracings **(F,G)**, and only the lateral V1 subbranch but not the medial V1 subbranch, in lateral V1 tracings **(J,K)**. **(D,H,L)** A negative control of the immunostainings is provided to distinguish tracer signal from endogenous pigment. The nerves are outlined. Lateral is right, dorsal is up. Abbreviations: d, dorsal; l, lateral; m, medial; v, ventral. Scale bars: **(D)** (for **A–D**), 50 μm; **(H)** (for **E–H**), 50 μm; **(L)** (for **I–L**), 50 μm.

**FIGURE 3:**
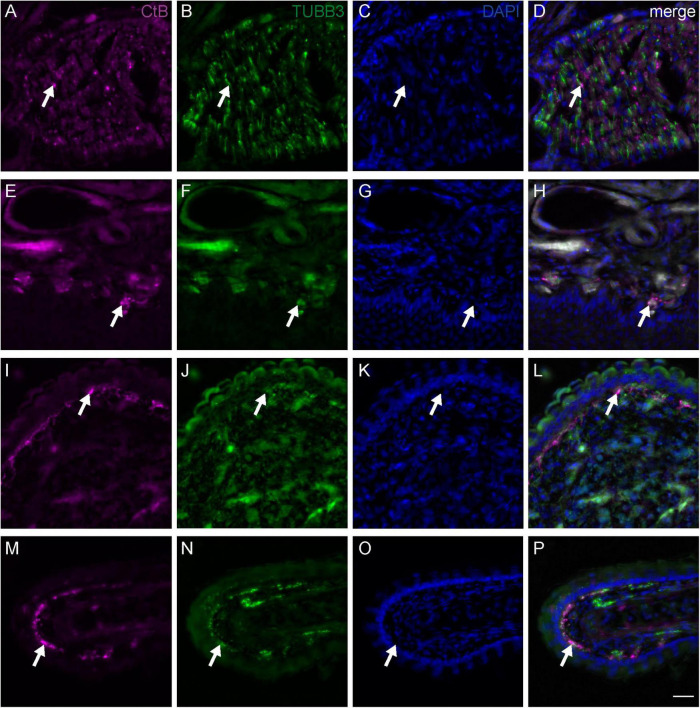
Fiber terminals of the medial subbranch of the ophthalmic branch of the trigeminal nerve (V1) in the upper beak of Eurasian blackcaps. Representative frontal slices of the upper beak of Eurasian blackcaps labeled against the neuronal tracer Cholera toxin B subunit (CtB; magenta) injected into the medial V1 subbranch, the general neuronal marker Tubulin beta 3 (TUBB3; green), and the cell nuclei marker DAPI (blue). **(A–D)** The CtB-traced nerve fibers in the medial V1 subbranch regionally colocalized with TUBB3 (arrows), thus verifying the tracing of neuronal tissue. **(E–H)** Fiber terminals (representative examples indicated by arrows) of the medial V1 subbranch were mainly located in the ventral subepidermis, **(I–L)** in the bulges of the ventral part of the nasal cavity and **(M–P)** in outgrowths of the septum. The non-100% overlap between TUBB3 and CtB is due to methodological limitations, mainly: (1) neuronal tracing will never label all nerve fibers and (2) some fibers will evade immunolabeling due to the less sensitive immunofluorescent labeling method as compared to the DAB staining method (e.g., used in Figure 2). Lateral is left, dorsal is up. Scale bars: **(P)** (for **A–P**), 20 μm.

**FIGURE 4:**
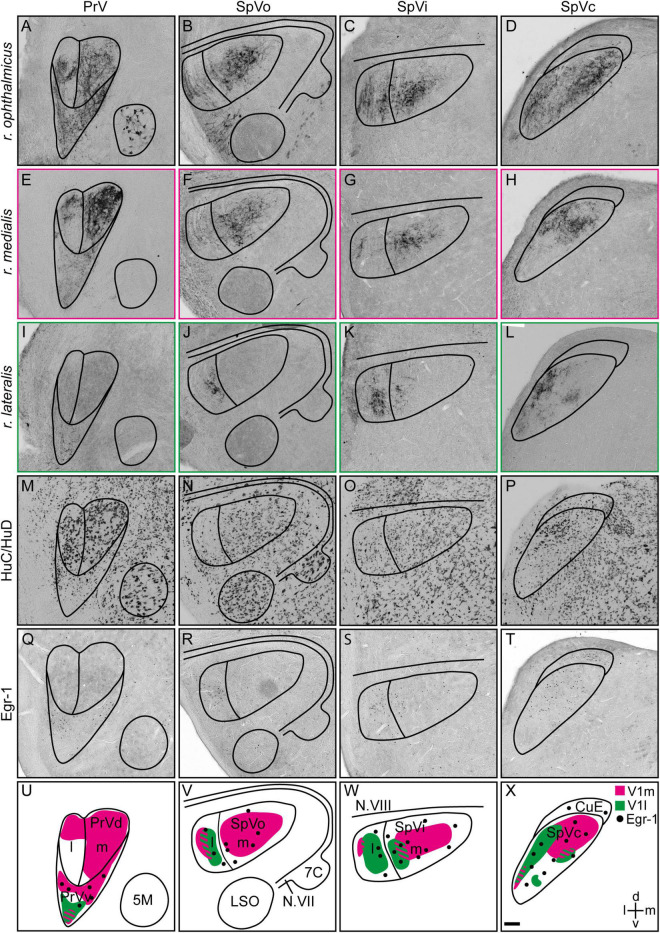
Projections of the medial and lateral subbranches of the ophthalmic branch of the trigeminal nerve (V1) in the trigeminal brainstem complex. **(A–D)** Anterograde projections labeled with Cholera toxin B subunit of the entire V1 (*r. ophthalmicus*), **(E–H)** the medial V1 (*r. medialis*) and **(I–L)** the lateral V1 subbranches (*r. lateralis*) in PrV, SpVo, SpVi, and SpVc, respectively, are shown on frontal brain slices. **(M–P)** HuC/HuD was used to define subcompartments within the trigeminal brainstem complex based on neuronal soma sizes. **(Q–T)** Magnetic-field induced neuronal activation in the trigeminal brainstem complex of Eurasian blackcaps was visualized using Egr-1. **(U–X)** Schematic illustrations of the innervation patterns of the medial (magenta) and lateral (green) V1 subbranches in PrV, SpVo, SpVi, and SpVc are shown. Regions of innervation overlap are striped. Magnetically activated subcompartments are indicated by black dots. Representative images are shown in panels **(A–T)**. Lateral is left, dorsal is up. Abbreviations: 5M, motor nucleus of the trigeminal nerve; 7C, facial nucleus; CuE, external cuneate nucleus; d, dorsal; Egr-1, early growth response protein 1; l, lateral; LSO, lateral superior olivary nucleus; m, medial; N.VII, facial nerve; N.VIII, vestibulo-cochlear nerve; SpV, spinal sensory trigeminal brainstem nucleus; SpVc, caudal part of spinal sensory trigeminal brainstem nucleus; SpVi, interpolar part of spinal sensory trigeminal brainstem nucleus; SpVo, oral part of spinal sensory trigeminal brainstem nucleus; PrV, principal sensory trigeminal brainstem nucleus; PrVd, dorsal part of principal sensory trigeminal brainstem nucleus; PrVv, ventral part of principal sensory trigeminal brainstem nucleus; V1, ophthalmic branch of the trigeminal nerve; V1l, lateral subbranch of the ophthalmic branch of the trigeminal nerve; V1m, medial subbranch of the ophthalmic branch of the trigeminal nerve; v, ventral. Scale bar: **(X)** (for **A–X**), 100 μm.

The permanent 3′3-diamino-benzidine (DAB) staining method was used to map the detailed course of the nerve (the basis of Figures 1, 2, 4) because this method is more sensitive and detects more fibers than immunofluorescence staining methods. For the double stainings (Figure 3); however, we had to use the less-sensitive fluorescence staining method.

For the permanent DAB staining, parallel series of brain or beak slices were stained together. Brain slices were stained free-floating, beak slices were stained on glass slides. Slices were washed three times for 10 min with Tris-buffered saline (TBS; pH 7.6). Endogenous peroxidases were saturated with 0.3% hydrogen peroxide for 30 min. After three times of washing for 10 min each in TBS, unspecific binding sites were blocked with 10% normal donkey serum (NDS; Antibodies-online, Aachen, Germany) dissolved in TBS containing 0.3% Triton-X100 (TBS-T; Carl Roth, Karlsruhe, Germany) for 30 min. Slices were incubated either with a primary polyclonal rabbit anti-CtB antibody (1:1000 in 5% NDS in TBS-T; C3062, lot. 045M4864V, Sigma-Aldrich, St. Louis, MO, United States, RRID: AB_258833) overnight at 4°C, with the general neuronal marker monoclonal mouse anti-HuC/HuD antibody (1:500 in 5% NDS in TBS-T; A21272, lot. 1963099, Invitrogen, Carlsbad, CA, United States, RRID: AB_2535822) overnight at 4°C, or with a polyclonal rabbit anti-Egr-1 antibody (1:1000 in 5% NDS in TBS-T; SC-189, lot. A2516, Santa Cruz Biotechnology, Dallas, TX, United States, RRID: AB_2231020) for 72 h at 4°C.

After washing three times for 10 min each in TBS, slices were incubated with a biotinylated secondary antibody (1:500 for brain slices, 1:200 for beak slices in TBS-T; PK-4002, PK-6101, Vector Laboratories, Burlingame, CA, United States) for 120 min at room temperature. After three washing steps for 10 min each in TBS-T, the slices were incubated in an avidin-coupled peroxidase complex (according to the manufacturer’s instructions, PK-4002, PK-6101, Vector Laboratories, Burlingame, CA, United States) for 60 min at room temperature. After two washing steps of 10 min each in TBS and inactivation of ongoing reactions in 0.1 M sodium acetate for 5 min, peroxidase activity was visualized under continuous visual inspection for 20 min using a 3′3-diamino-benzidine (Acros Organics, Fair Lawn, NJ, United States) reaction using glucose oxidase (0.2% in glycerol and distilled water; Sigma-Aldrich, St. Louis, MO, United States) instead of hydrogen peroxide (Shu et al., 1988). The reaction was stopped by incubating the slices in 0.1 M sodium acetate for 5 min. The stained brain slices were mounted on glass slides (Menzel SuperFrost ^®^ Plus, Thermo Fisher Scientific, Waltham, MA, United States). Brain and beak slices were dehydrated in a graded series of alcohol (70% ethanol, 96% ethanol, isopropanol, twice xylene) and cover-slipped with Eukitt (Sigma-Aldrich, St. Louis, MO, United States). Negative controls were performed on parallel beak slices omitting the primary antibody to distinguish endogenous pigment epithelium from the substrate staining. Brain and beak slices were imaged using light microscopy (Zeiss Axio Scan.Z1, Oberkochen, Germany, objective: 10× Plan-Apochromat, 0.45 NA). Contrast was adjusted with identical settings using the function *Enhance contrast* of ImageJ (Version: 1.53f51, NIH, Bethesda, MD, United States; RRID: SCR_003070; Schindelin et al., 2012).

For fluorescent immunohistochemical stainings, beak slices were washed three times in TBS for 10 min each, and unspecific binding sites were blocked with 10% NDS (in TBS-T; Antibodies-online, Aachen, Germany) for 1 h. Slices were incubated with a polyclonal rabbit anti-CtB antibody (1:1000 in 5% NDS in TBS-T; C3062, lot. 045M4864V, Sigma-Aldrich, St. Louis, MO, United States, RRID: AB_258833) and a monoclonal mouse anti-Tubulin beta 3 (TUBB3) antibody (1:500 in 5% NDS in TBS-T; 801201, lot. B249869, BioLegend, San Diego, CA, United States, RRID: AB_10063408) overnight at 4°C. Slices were washed three times 5 min each in TBS before the TUBB3 signal was enhanced by incubating the slices with a secondary biotinylated horse anti-mouse antibody (1:500 in TBS-T; PK-4002, Vector Laboratories, Burlingame, CA, United States) for 90 min at room temperature. After three times of 5-min washing each in TBS, primary antibodies were detected with Alexa-conjugated secondary antibodies (each 1:500 in TBS-T; Streptavidin Alexa Fluor 488 conjugate, S32354, Invitrogen, Carlsbad, CA, United States; Alexa Fluor 568 donkey anti-rabbit, ab175692, Abcam, Cambridge, United Kingdom) on incubation for 90 min at room temperature. Slices were cover-slipped with 4′,6-Diamidin-2-phenylindol (2 μg/ml, Carl Roth, Karlsruhe, Germany) dissolved in DABCO (Carl Roth, Karlsruhe, Germany) and Mowiol (Carl Roth, Karlsruhe, Germany). Negative controls were performed on parallel beak slices omitting the primary antibodies. Beak slices were imaged using fluorescence microscopy (Leica DM6 B, Wetzlar, Germany, objective: 20× HC PL FLUOTAR, 0.50 NA). With ImageJ (Version: 1.53f51, NIH, Bethesda, MD, United States; RRID: SCR_003070; Schindelin et al., 2012) background subtraction was done, the contrast was adjusted using the function *Enhance contrast*, and the maximum projection of each stack was used, all with identical settings.

### Anatomical Mapping

To reconstruct the course of V1 and its subbranches, a series of beak slices stained against CtB were systematically aligned and normalized based on hand drawings in a coordinate grid using a pen display (three animals; Wacom Cintiq 21UX, Wacom, Düsseldorf, Germany) and the Adobe Illustrator 25.4.1 software (Adobe Systems Software, Dublin, Ireland, RRID: SCR_010279). The course of V1 and its subbranches was translated into 2D reconstructions, one depicting the course of the subbranches as a side view (Figure 1A) showing the dorsoventral coordinates of the nerve subbranches, and one as viewed from above (Figure 1B) to show the mediolateral coordinates. Anatomical boundaries within the trigeminal brainstem complex were defined based on previous studies on the known restricted expression of the immediate early gene Egr-1 and on morphometric features using the general neuronal marker HuC/HuD (Heyers et al., 2010; Lefeldt et al., 2014; Elbers et al., 2017; Faunes and Wild, 2017b; Kobylkov et al., 2020).

## Results

### Methodological Considerations

During the experiments, we took the greatest care to make our tracings as replicable as possible, i.e., by accessing the nerve subbranches at the same location within the beak and injecting the same amounts of tracer. Nevertheless, due to the predictable methodological limitations of this technique, we observed slightly different innervation patterns between single specimens. However, all our core findings were observable in all cases, and for the figures, we carefully compared all “cases” and chose the most representative ones to appear in our manuscript.

### V1 Subbranch Course in the Upper Beak

To identify the exact course and the distal terminations of V1 within the upper beak, we either selectively traced the medial or the lateral V1 subbranch in the night-migratory songbird Eurasian blackcap (Figures 1A, 2B,C,F,G,J,K; magenta and green). For comparison, we traced the entire V1 at a level where the medial and lateral V1 subbranch had already fused into one nerve (Figures 1A, 2A,E,I; black). We confirmed the successful selective tracing by analyzing the tracing patterns in beak slices. At the caudal end of the upper beak beyond the point where the medial and lateral V1 subbranches are fused, only selective V1 parts were labeled by medial or lateral V1 tracings (Figures 2A–D). In rostral parts of the upper beak either only the medial V1 subbranch was labeled by V1 medial tracings or the lateral V1 subbranch by lateral V1 tracings (Figures 2E–L). Furthermore, double immunofluorescence stainings confirmed that we indeed traced neuronal tissue, depicted as a partial colocalization of the tracer CtB (magenta) and the neurofilament marker TUBB3 (green; Figures 3A–D). The section level, in which V1 exits the orbit, was set as the anterior–posterior zero coordinate (approximately A 0 mm), and the tip of the beak was set as the dorso-ventral zero coordinate (approximately A 16 mm).

The ophthalmic branch of the trigeminal nerve leaves the orbit rostrally through the ophthalmic foramen, medially accompanied by the olfactory nerve (Figures 1A,B, 2A–D). V1 fuses with parts of the conchal lobes, and at approximately A 2.5 mm it rostrally bifurcates into its medial (*r. medialis*; magenta) and lateral subbranches (*r. lateralis*; green; Figures 1A–C).

The medial V1 subbranch turns ventromedial to find its way through the nasal septum and reaches the base of the upper beak at approximately A 3.8 mm. On its way toward the tip of the upper beak, it separates into two further subbranches approximately at the level of the nostrils (A 9.9 mm; Figures 1A–C, pink and violet). These subbranches likely represent the *r. pre-maxillaris ventralis*, the main continuation of the *r. medialis* (Figures 1C, 2I–L). Both, *r. pre-maxillaris ventralis* subbranches run in parallel further rostrally. Its larger portion runs laterally (pink) and can be tracked almost up to the tip of the beak (approximately A 15.7 mm; Figures 1A–C). The *r. pre-maxillaris ventralis* innervates the mucosa of the rostral half of the palate and the tip of the upper beak (Bubien-Waluszewska, 1981). Distal fiber terminals of the medial V1 subbranch are mainly located in the ventral subepidermis (Figures 3E–H), in the bulges of the ventral part of the nasal cavity (Figures 3I–L), and in outgrowths of the septum (Figures 3M–P; see Figure 1D for anatomical terminology). The *rr. nasales interni* and the *r. pre-maxillaris dosalis* of the *r. medials* described in chicken (Bubien-Waluszewska, 1981) could not be found in blackcaps (Figure 1C).

After separating from the medial V1 subbranch, the lateral V1 subbranch runs rostrally at approximately the same dorso-ventral level as the entire V1. At approximately A 5.5 mm, it splits up into two further subbranches, likely resembling the *rr. nasales interni* [Figures 1A–D; light and dark green; very similar to what was shown in chicken (Bubien-Waluszewska, 1981)]. Both initially take their course inside the nasal bone, while the dorsal subbranch (light green) exits the nasal bone again and takes its course on its surface (Figures 1A,B, 2E–H). Both can be tracked until the opening of the nostrils become apparent (at A 9.9 mm; Figures 1A,B). The *rr. nasales interni* are known to innervate the mucosa of the lateral nasal cavity (Bubien-Waluszewska, 1981). Two further subbranches known from chicken (Bubien-Waluszewska, 1981), i.e., the *rr. frontales* and the *rr. palpebrales rostro-dorsales*, could not be identified in blackcaps (Figure 1C).

### V1 Subbranch Projections to the Trigeminal Brainstem Complex

To identify the trigeminal brainstem terminations of the medial and lateral V1 subbranches, we selectively injected neuronal tracer into the respective subbranches (Figures 1A, 4E–L; magenta and green) and analyzed the anterograde innervation patterns in the PrV and SpV in Eurasian blackcaps. Additionally, neuronal tracings of the entire V1 were performed for comparison of the innervation patterns (Figures 4A–D; black). To define the neuroanatomical boundaries of the respective brain areas, a general neuronal marker (HuC/HuD) was used (Figures 4M–P). The dorsal and ventral parts of PrV were previously shown to be distinguishable based on their respective soma sizes (Figure 4M; Kobylkov et al., 2020).

Medial subbranch tracings revealed terminations in both the medial and lateral parts of the dorsal part of PrV and a termination field in the magnetically activated ventral part of PrV (Figures 4E,U; magenta), depicted by the expression of the immediate early gene Egr-1 on parallel brain slices (Figures 4Q,U). Lateral V1 subbranch tracings revealed a small region with fiber terminals in the ventral tip of PrV (Figures 4I,U; green). The termination field of the medial and lateral V1 subbranches partially overlapped in the ventral part of PrV (Figure 4U).

In the SpV, medial V1 subbranch tracings resulted in the labeling of fiber terminals mainly in all medial parts of SpV and in small parts of the lateral part of SpV (Figures 4F–H,V–X; magenta). Lateral V1 subbranch tracings labeled fiber terminals mainly in lateral parts of SpV. However, the termination field gradually moved medial toward caudal SpV levels (Figures 4J–L,V–X; green). The termination fields of the medial and lateral V1 subbranches in SpV partially overlapped, while generally conserving the topographic innervation (Figures 4V–X).

## Discussion

Previous neurobiological studies have suggested an involvement of the trigeminal brainstem complex in processing magnetic information. More specifically, the ventral part of PrV and parts of SpV were shown to display magnetic field-induced, V1-mediated, neuronal activation (Heyers et al., 2010; Lefeldt et al., 2014; Elbers et al., 2017). Recently, we identified a previously unknown brain pathway, with which magnetic information from the ventral part of PrV is most likely being sent to the telencephalic frontal nidopallium (Kobylkov et al., 2020). Furthermore, physical, geographical, and virtual magnetic displacements have indicated that V1, which innervates the trigeminal brainstem complex, is essential for providing magnetic positional information (Kishkinev et al., 2013; Pakhomov et al., 2018).

All aforementioned studies mapped V1 connectivities and hypothesized on its potential functions based on tracings and ablations of the entire V1. In contrast, this study provides the first precise neuroanatomical data from a night-migratory songbird on the exact course of V1 down to the level of its subbranches and their distal terminals and its proximal terminations in the trigeminal brainstem complex. The dorsal part of PrV, known to be involved in processing somatosensory information, is exclusively innervated by the medial V1 subbranch (Figures 4E,U). The magnetically activated ventral part of PrV receives input from both the medial and lateral V1 subbranches (Figures 4E,I,Q,U). In SpV, the topographic innervation is mostly conserved, i.e., with the majority of fibers terminating in the medial parts belonging to the medial V1 subbranch, while the lateral V1 subbranch mainly terminates in lateral parts of SpV (Figures 4F–H,J–L,V–X).

In addition, we mapped the detailed course of each of the V1 subbranches up to its distal fiber terminals in the upper beak of a night-migratory songbird. The medial V1 subbranch runs along the ventral part of the upper beak to innervate its subepidermal layers and the septal and ventral parts of the mucosa within the nasal cavity (Figures 1, 2, 3). The lateral V1 subbranch runs along the dorso-lateral part of the beak innervating superficial tissue such as the skin of the forehead, the upper eyelid, the conjunctiva of the nasal commissure, and major parts of the mucosa of the nasal cavity (Figures 1, 2; Bubien-Waluszewska, 1981). Comparing the currently available literature with the respective courses of the tracer-labeled subbranches, our findings generally reflect the described anatomy of V1 and its subbranches in chicken (Bubien-Waluszewska, 1981), suggesting that the neuroanatomy of V1 is mainly conserved across the avian clade.

Do the restricted innervation patterns allow us to assign processing of information of a certain quality (somatosensation, magnetoreception, and/or both) to each of the V1 subbranches? Our data indicate that the medial V1 is most likely responsible for mediating somatosensory information from the upper beak since it is the only V1 subbranch that exclusively innervates the dorsal part of PrV (Figures 4E,U). The dorsal part of PrV is known to form the origin of fibers carrying somatosensory information (mechanoreception, proprio-, thermo-, chemo-, and nociception) to the telencephalic Nucleus basorostralis (Wild et al., 1985; Kobylkov et al., 2020). This fact is supported by the location of medial V1 fiber terminals in the ventral subepidermal layer of the upper beak, the septum, and ventral parts of the nasal cavity (Figures 3E–P). These beak parts contain a dense multisensory innervation network, which birds will almost certainly consult when performing somatosensory-mediated behaviors with their beaks such as feeding, grooming, climbing, nest building, hatching, and exploring the environment (Wild, 2015). The fact that additional medial V1 fibers terminate in the magnetically ventral part of PrV and that many medial V1 fibers terminate in magnetically activated parts of SpV (Figures 4E–L,Q–X) suggests that the medial V1 subbranch could also be involved in the processing of magnetic information. The lateral V1 subbranch solely terminates in a small portion of the magnetically activated ventral part of PrV (Figures 4I,Q,U), which would point toward a potential primary involvement in the processing of magnetic information. However, in the SpV, only some parts of the lateral V1 fibers terminate in magnetically activated SpV portions (Figures 4J–L,R–T,V–X), which would mean that some but not all lateral V1 fibers could be also involved in magnetoreception. Furthermore, some lateral subbranch neurons could still be involved in conveying somatosensory information to the dorsal part of PrV *via* interneurons passing on information from SpV to PrV.

Based on our expectation that any magnetic sensor must be located within the proximity of any of the V1 subbranches, do our data allow us to narrow in on the exact location of any magnetic sensor? At present, the answer is no since our data show that both subbranches innervate different magnetically activated parts of the trigeminal brainstem complex to varying degrees (Figure 4). Thus, based on our findings, it seems reasonable that both the medial as well as the lateral V1 subbranch might be involved in mediating magnetic information. Apart from the fact that assigning a specific function to a specific nerve subbranch is certainly too simplistic, any functional analyses based on purely anatomical data in our study are further hampered by the simple fact that (1) the lateral V1 subbranch is smaller than its medial counterpart (Figure 2), i.e., it consists of fewer neurons and, consequently, has both a smaller distal and proximal dendritic innervation field, (2) the magnetically activated ventral part of PrV does seem to contain a mix of neurons encoding for both magnetic and somatosensory information, since neuronal tract tracings from the connected telencephalic frontal nidopallium labeled only 15% of the magnetically activated neurons in the ventral part of PrV (Kobylkov et al., 2020), and, (3) we cannot rule out any possible interneuronal connectivities within the respective trigeminal brainstem subcompartments.

Only a highly elaborate correlation to function approach, e.g., the analysis of magnetic field-induced neuronal activation and/or a behavioral displacement study after selective ablation of either the medial or lateral V1 subbranch could give further evidence about any specific involvement of one of the V1 subbranches in magnetoreception. However, given the complicated innervation patterns seen here, the estimated number of experimental animals needed to reach a clear conclusion, if any exists that could be obtained from such studies, would exceed our ethical limits, and we have therefore chosen not to perform such studies. In conclusion, the elusive trigeminal-based magnetic sensors could be located anywhere along the entire dendritic field of both V1 subbranches.

## Data Availability Statement

The original contributions presented in the study are included in the article/supplementary material, further inquiries can be directed to the corresponding author.

## Ethics Statement

The animal study was reviewed and approved by the Animal Care and Use Committees of the Niedersächsisches Landesamt für Verbraucherschutz und Lebensmittelsicherheit (LAVES, Oldenburg, Germany, Az.: 33.19-42502-04-15/1865; 33.19-42502-04-20/3492; and 33.8-42502-04-17/2724).

## Author Contributions

DH and HM designed the research. KH, IM, LW-M, BL, AZ, and DH performed experiments. KH, IM, LW-M, and DH analyzed the data. HM provided facilities. KH and DH wrote the first draft of the manuscript, which all co-authors commented on. All authors contributed to the article and approved the submitted version.

## Conflict of Interest

The authors declare that the research was conducted in the absence of any commercial or financial relationships that could be construed as a potential conflict of interest.

## Publisher’s Note

All claims expressed in this article are solely those of the authors and do not necessarily represent those of their affiliated organizations, or those of the publisher, the editors and the reviewers. Any product that may be evaluated in this article, or claim that may be made by its manufacturer, is not guaranteed or endorsed by the publisher.
